# Tetrahedral framework nucleic acids for improving wound healing

**DOI:** 10.1186/s12951-024-02365-z

**Published:** 2024-03-16

**Authors:** Wanqing Zou, Jing Lu, Luyong Zhang, Duanping Sun

**Affiliations:** 1 Guangdong Provincial Key Laboratory of Pharmaceutical Bioactive Substances, Center for Drug Research and Development, Guangdong Pharmaceutical University, Guangzhou, 510006 Guangdong China; 2 Key Specialty of Clinical Pharmacy, The First Affiliated Hospital of Guangdong Pharmaceutical University, Guangzhou, 510699 Guangdong China; 3 National and Local United Engineering Lab of Druggability and New Drugs Evaluation, School of Pharmaceutical Sciences, Sun Yat-Sen University, Guangzhou, 510006 Guangdong China; 4 Jiangsu Key Laboratory of Drug Screening, China Pharmaceutical University, Nanjing, 210009 Jiangsu China

**Keywords:** Tetrahedral framework nucleic acids, Wound healing, DNA nanomaterials, Tissue regeneration

## Abstract

Wounds are one of the most common health issues, and the cost of wound care and healing has continued to increase over the past decade. In recent years, there has been growing interest in developing innovative strategies to enhance the efficacy of wound healing. Tetrahedral framework nucleic acids (tFNAs) have emerged as a promising tool for wound healing applications due to their unique structural and functional properties. Therefore, it is of great significance to summarize the applications of tFNAs for wound healing. This review article provides a comprehensive overview of the potential of tFNAs as a novel therapeutic approach for wound healing. In this review, we discuss the possible mechanisms of tFNAs in wound healing and highlight the role of tFNAs in modulating key processes involved in wound healing, such as cell proliferation and migration, angiogenesis, and tissue regeneration. The targeted delivery and controlled release capabilities of tFNAs offer advantages in terms of localized and sustained delivery of therapeutic agents to the wound site. In addition, the latest research progress on tFNAs in wound healing is systematically introduced. We also discuss the biocompatibility and biosafety of tFNAs, along with their potential applications and future directions for research. Finally, the current challenges and prospects of tFNAs are briefly discussed to promote wider applications.

## Introduction

The largest organ in the human body and the entire defense system is the skin, which makes up approximately 15% of body weight [1]. To maintain homeostasis, the many distinct cell types that make up the large and complex human skin interact with one another in a highly coordinated way [2]. Due to prolonged exposure, the skin is exposed to a variety of external stimuli. The most common skin injuries are caused by acute trauma, chronic trauma, or surgical procedures [3]. Oxidative stress, decreased angiogenesis, increased expression of proinflammatory cytokines, and bacterial infection all contribute to chronic inflammation in the wound microenvironment [4–6]. Overall, wound infection is characterized by the colonization of bacteria and other microbes, which can lead to delayed wound healing or cause the lesion to deteriorate.

Wounds are susceptible to infection by bacteria and other microorganisms, especially if the external environment is unclean or personal hygiene is poor. Infection can slow the wound healing process and may lead to more serious complications [7]. The main objective of wound care is to avoid serious infections, promote wound healing, and reduce scarring and pain [8]. Clinical professionals have struggled with wound healing for a long time, and new materials and techniques are desperately needed [9]. Although there are many nanoscale materials to choose from, in many cases, their size and shape are imprecise, they can only be distributed within a certain range, and it is difficult to achieve precise control. In contrast, framework nucleic acids self-assembled through DNA programming have the characteristics of single molecular weight, precise structure, controllable size and shape, and adjustable mechanical properties, which provide advanced materials for regulating cell transmembrane processes and organ targeting in vivo with tools [10–12]. For instance, oligonucleotides can be organically combined with tFNAs during the solid-phase synthesis of scaffold strands. Therefore, tFNAs are capable of loading therapeutic nucleic acids to promote wound healing [13]. tFNAs also enable site-specific organization of small or macromolecules or nanoparticles with nanometer precision [14–17].

tFNAs are pyramidal three-dimensional nanostructures formed by the complementary pairing of four single-stranded DNAs [18, 19]. tFNAs are a novel class of DNA nanomaterials that have drawn significant interest because of their simple synthesis, high availability, stable structure, and variety of applications [20, 21]. tFNAs have a range of unique properties, including structural rigidity, tunable sizes, and the ability to engineer specific sequences into the edges or anchor them to structures [22]. The potential for wound healing of tFNAs may be attributed to their various molecular functions, such as inhibition of apoptosis, promotion of cell division, proliferation, and migration, inhibition of expression of anti-inflammatory factors, reduction of extracellular polysaccharides and biofilm, and reduction of reactive oxygen [23].

In summary, tFNAs have many merits, hold tremendous potential for treating wound infection and are currently a hot research area in wound healing. While several excellent reviews on tFNAs have been published [24–28], reviews focusing on tFNAs for wound healing applications are urgently needed. Therefore, it is necessary to summarize the latest advances to provide insights into the development trends of tFNAs in this research area. In this review, we summarize the latest achievements of tFNAs with promising applications from different perspectives, highlighting the characteristics of tFNAs and elaborating on their possible mechanisms for promoting wound healing and the progress of their applications. In addition, biosafety issues in their practical applications are discussed. Finally, a brief conclusion and discussion of the existing challenges and further perspectives are given.

## Wound healing process

**Fig. 1:**
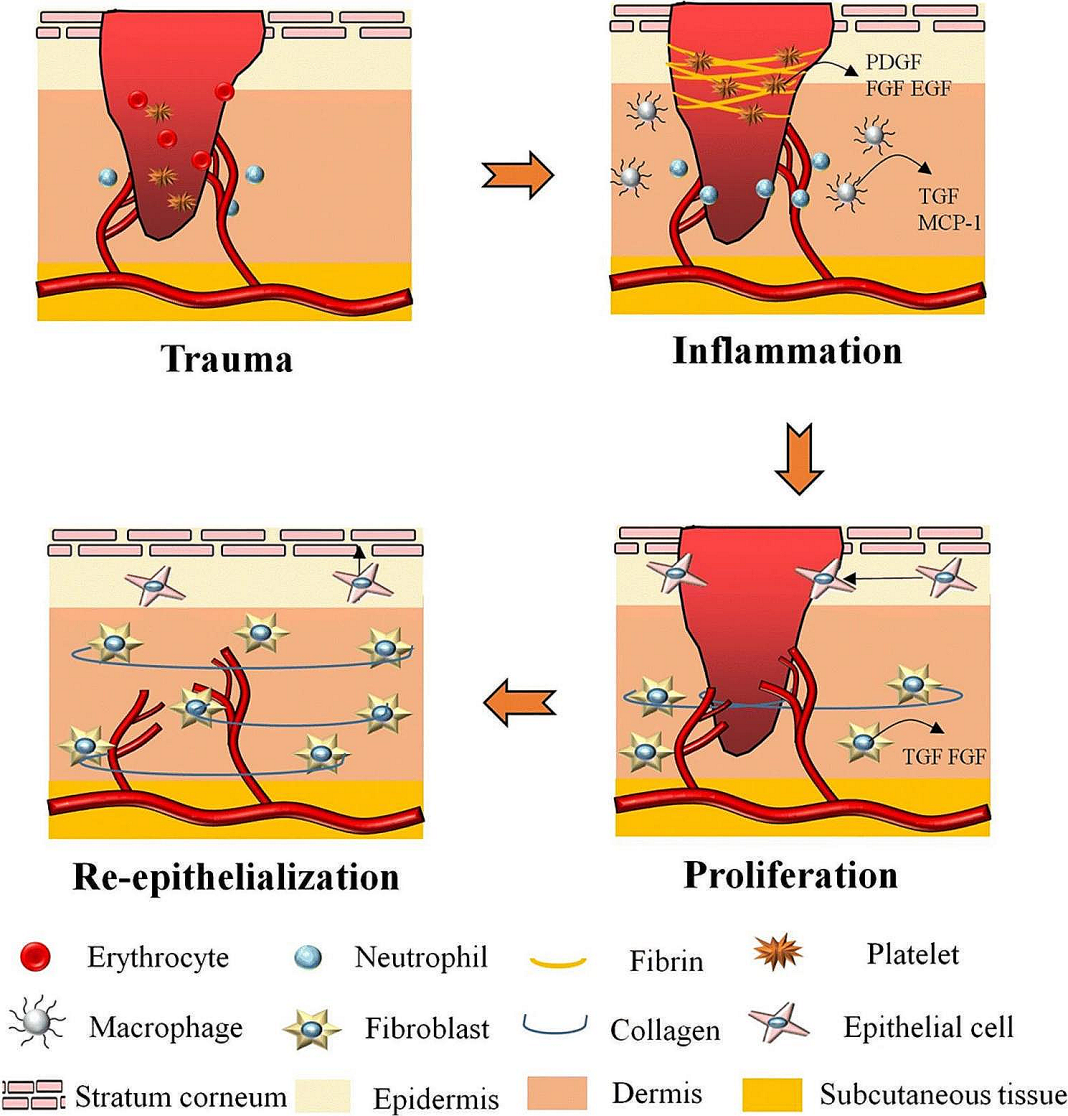
A diagram of the wound healing process. PDGF: platelet-derived growth factor; FGF: fibroblast growth factor; EGF: epidermal growth factor; TGF: transforming growth factor; MCP-1: monocyte chemotactic protein 1. Reprinted with permission from Ref. [8]

Wound healing involves four distinct phases: hemostasis, inflammation, proliferation, and dermal remodeling. These phases entail the intricate and coordinated interactions of various cell types and growth factors, as shown in Fig. 1 [29].

Bleeding usually starts immediately after an injury. Vasoconstriction, primary hemostasis, and secondary hemostasis are the three main processes by which blood eliminates bacteria and other pathogens from the wound. Platelets and fibrinogen are the primary cellular and matrix components of this process, respectively. Vasoconstriction of the blood vessel wall is the first step of hemostasis after injury. Thus, primary and secondary hemostasis operate via two connected and concurrent pathways. Finally, the platelet plug and fibrin mesh eventually combine to form a thrombus, which stops the bleeding and maintains homeostasis. This produces a transient scaffold that enables the infiltration of healing cells [30]. After the inflammatory phase, the blood vessels become more permeable, enabling immune cells and enzymes to reach the injury site. Neutrophils predominantly accumulate during the first three days, followed by monocytes. Reactive oxygen species (ROS) and matrix metalloproteinases (MMPs) are released by neutrophils and clear wound debris and any bacterial contamination simultaneously [31, 32]. Once at the injury site, monocytes undergo differentiation to become macrophages, which phagocytize dead neutrophils and nonviable tissue fragments. Furthermore, the release of numerous growth factors and cytokines triggers the recruitment of fibroblasts, keratinocytes, and endothelial cells to repair damaged vessels [33].

When the proliferative phase begins, which can continue for up to 14 days, these cells intensify the inflammatory response and cause the creation of granulation tissue. Angiogenesis, epithelialization, granulation tissue, and collagen deposition are all included in this phase [34, 35]. To produce granulation tissue and deposit extracellular matrix, endothelial cell proliferation and angiogenesis are necessary (ECM). This is encouraged by fibroblast growth factor (FGF) and vascular endothelial growth factor (VEGF) [36]. In the early stages, fibroblasts are the predominant cell type. Part of this population transforms into myofibroblasts, which cause wound closure. ECM components are secreted by fibroblasts and are the basis for skin healing [37]. Remodeling is the third step of wound healing. All of these processes are activated after the injury occurs and stop during this phase. The majority of endothelial cells, macrophages, and myofibroblasts either undergo apoptosis (programmed cell death) or leave the wound, leaving a cell-free mass mainly composed of collagen and other extracellular matrix proteins. Skin integrity and homeostasis are likely continuously regulated by the interaction between the epithelium and the mesenchyme [38]. Additional feedback loops must be in place to maintain other skin cell types. Additionally, the acellular matrix actively remodels itself throughout 6 to 12 months, switching from having a backbone made primarily of type III collagen to one made primarily of type I collagen [39]. In this case, matrix metalloproteinases, commonly secreted by macrophages, fibroblasts, and endothelial cells, function to strengthen the repaired tissue [40].

## tFNAs in wound healing

Turberfield et al. [41] first proposed tetrahedral DNA nanostructures (TDNs), also known as “tetrahedral framework nucleic acids” (tFNAs), in 2005. Since then, numerous research teams have focused on tFNAs and their applications, and numerous fresh design approaches for tFNAs have been published in academic papers [42]. tFNAs are one of the simplest DNA polyhedrons with strong structural stability since each face is a complete triangle [43].

The majority of tFNAs use a standard synthesis process and have a homogeneous shape and size [44]. Four single nucleic acids make up the fundamental tetrahedral nanostructure, which uses the complementary pairing principle to self-assemble into a tetrahedral 3D structure [45, 46]. Each single-stranded DNA was designed to include three sections of complementary sequences to the other three strands [47, 48]. Therefore, each strand that binds to the other three nucleic acids produces one side of the tetrahedron, and one of the three fragmented sequences that are combined in pairs creates the double-helix structure on the edge of the tetrahedron [49]. The synthesis of tFNAs involves two key steps: the design of individual nucleic acid strands and assembly into a tetrahedral structure [49–51]. First, suitable nucleic acid sequences are designed for the desired tFNA structure using computer-aided design (CAD) or DNA origami techniques [52]. These sequences typically consist of tens to hundreds of nucleotides, including complementary base pairs necessary for structural stability. Second, the assembly of individual nucleic acid strands is performed to transform them into the tetrahedral structure of tFNAs. Assembly can be achieved through self-assembly or externally guided methods [21, 53]. Self-assembly involves the mutual binding of individual nucleic acid strands based on complementary base pairing in an appropriate buffer solution to form the tetrahedral structure [54]. Externally guided methods utilize auxiliary DNA or RNA molecules as templates or guides, which interact with the individual nucleic acid strands through specific complementary pairing, facilitating the formation of the desired tFNA structure [55]. During the synthesis process, critical factors include optimal reaction conditions, buffer solution selection, and precise control of reaction time [56]. Additionally, surface modification is an important aspect of tFNA synthesis. By introducing modified nucleotides or chemical modifications, the stability, biocompatibility, and functionality of tFNAs can be modulated [57, 58]. We compared some information related to tetrahedral framework nucleic acid synthesis and listed it in Table 1.

**Table 1:** Synthesis methods and conditions of tFNAs

Synthesis method	Experimental method	Specific sequence (from 5’ to 3’)	Number of bases per single strand	Ref
Self-assembled	Heated at 95 °C for 2 min followed by incubation in ice bath	**S1** ACA TTC CTA AGT CTG AAA CAT TAC AGC TTG CTA CAC GAG AAG AGC CGC CAT AGT A **S2** SH-C6-TAT CAC CAG GCA GTT GAC AGT GTA GCA AGC TGT AAT AGA TGC GAG GGT CCA ATA C **S3** -SH-C6-TCA ACT GCC TGG TGA TAA AAC GAC ACT ACG TGG GAA TCT ACT ATG GCG GCT CTT C **S4** -SH-C6-TTC AGA CTT AGG AAT GTG CTT CCC ACG TAG TGT CGT TTG TAT TGG ACC CTC GCA T	73	[59]
Self-assembled	Heat up to 95 °C for 10 min then cool down to 4 °C for half an hour	**S1** ATTTATCACCCGCCATAGTAGACGTATCACCAG GCAGTTGAGACGAACATTCCTAAGTCTGAA **S2** ACATGCGAGGGTCCAATACCGACGATTACAGC TTGCTACACGATTCAGACTTAGGAATGTTCG **S3** ACTACTATGGCGGGTGATAAAACGTGTAGCAA GCTGTAATCGACGGGAAGAGCATGCCCATCC **S4** ACGGTATTGGACCCTCGCATGACTCAACTGCC TGGTGATACGAGGATGGGCATGCTCTTCCCG	63	[60]
Self-assembled	Heated to 95 °C for 4 min and cooled rapidly on ice for 30 min	**S1** CGCGACTTAGGTCCATAATCAAGGGGCCGGTG AGATGGGAGTGAACGGGTCTGG **S2** TAGCGTTAGGACAACGGAATCTCACCGGCCCC TTGATACGTGCGGGTCTGATAA **S3** TTATGGACCTAAGTCGCGAGTCCAGAATAAGG AACTTTATCAGACCCGCACGTA **S4** ACTCCAGACCCGTTCACTCCCTCCGTTGTCCAA CGCTAAG	54;54; 54;40	[61]
Self-assembled	Denatured at 95 °C for 5 min and then cooled to 4 °C for 1 h	**S1** CCAGGCAGTTGAGACGAACATTCCTAAGTCTG AAATTTATCACCCGCCATAGTAGACGTATCA **S2** CTTGCTACACGATTCAGACTTAGGAATGTTCGA CATGCGA GGGTCCAATACCGACGATTACAG **S3** GGTGATAAAACGTGTAGCAAGCTGTAATCGAC GGGAAGA GCATGCCCATCCACTACTATGGCG **S4** CCTCGCATGACTCAACTGCCTGGTGATACGAG GATGGGCA TGCTCTTCCCGACGGTATTGGAC	65;64 64;64	[62]
DNA origami	Annealed at 95 °C for 30 s, followed by a temperature decrease of 10 min/ °C until 65 °C, afterward a decrease of 20 min/ °C until 20 °C	**S1** GACTAAAGGAGGATATCATCGGACAG **S2** CGCATAACGGTCGAAAGGCCGATCGTCAC **S3** TATTCCGATATTAGCTTTAGCTT **S4** AGTTTCCATTAATTGTATCGGTATAGCGGGCT TTT	26;29 23;35	[63]
Enzymatic assembly	Heated to 90 °C for 5 min and then cooled down to 4 °C for 10 min	**S1** ACACTACGTCAGAACAGCTTGCATCACTGGTC ACCAGAGTA **S2** ACGAGCGAGTTGATGTGATGCAAGCTGAATGC GAGGGTCCT **S3** TCAACTCGCTCGTAACTACACTGTGCAATACT CTGGTGACC **S4** TCTGACGTAGTGTATGCACAGTGTAGTAAGGA CCCTCGCAT	41	[64]
Enzymatic assembly	Heated to 70 °C for 5 min and then cooled down to 4 °C for 1 h	**S1** AGGCAGTTGAGACGAACATTCCTAAGTCTGAA ATTTATCACCCGCCATAGTAGACGTATCACC **S2** TATTTTGTTATGTGTTATGTGTTATATTCAGACTT AGGAATGTTCGACATGCGAGGGTCCAATACCG **S3** CCCCCCCTAACCCCCCCTAACCCCCCCTAACC CCCCCATACTGTAATCGACGGGAAGAGCATGCCC ATCCACTACTATGGCGGGTGATAAA **S4** CCTCGCATGACTCAACTGCCTGGTGATACGAG GATGGGCATGCTCTTCCCGACGGTATTGGAC	63;67 91;63	[65]

**Fig. 2:**
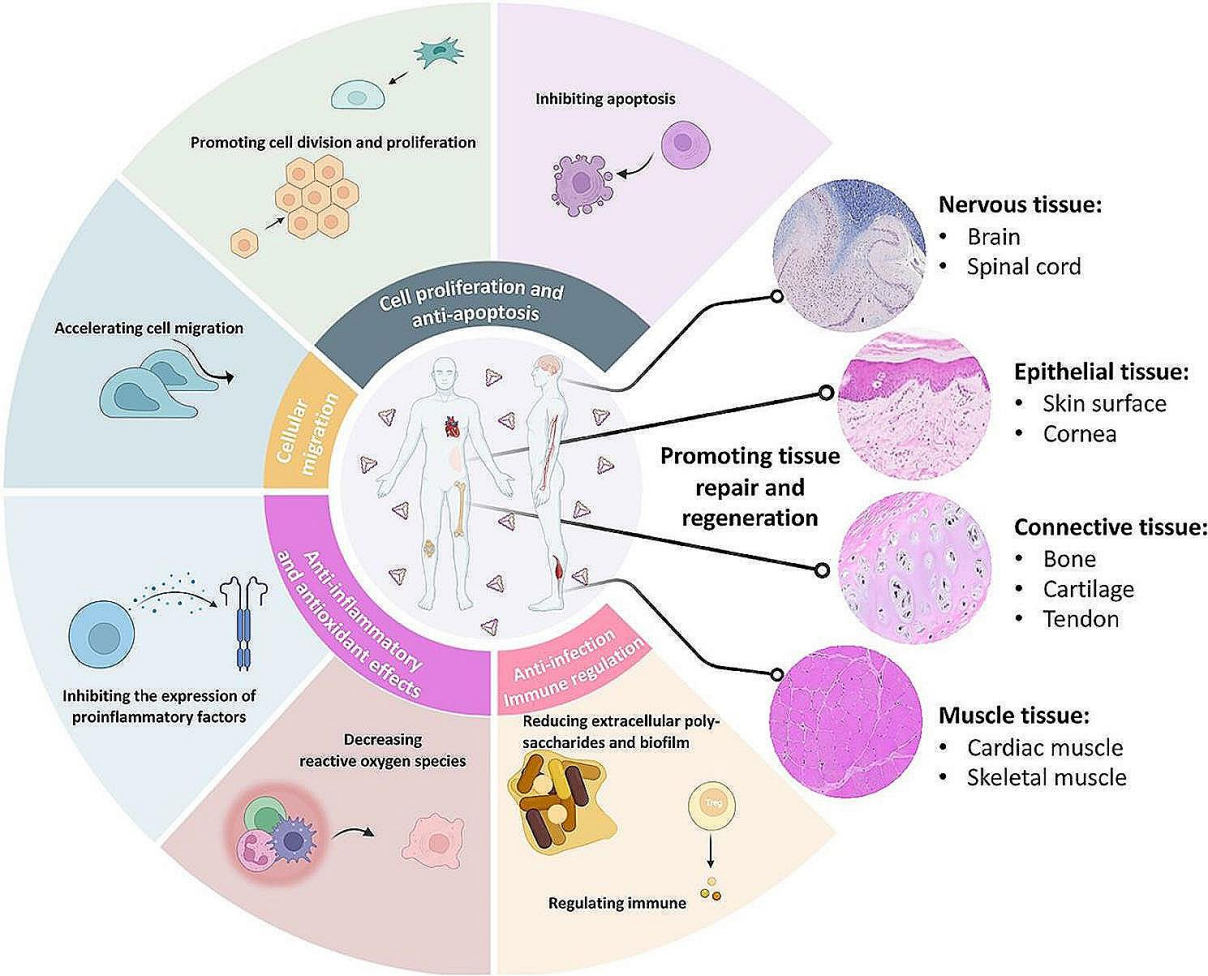
Tetrahedral DNA nanostructures play a vital role in the repair and regeneration of several tissues. Reprinted with permission from Ref. [23]

Compared to linear DNA or DNA octahedra, tFNAs are comparatively more stable [66]. tFNAs have the advantages of (i) tissue penetration and cellular uptake; (ii) better tolerance in biological environments; (iii) easy fabrication and high yield; and (iv) high yield and fast synthesis speed. It has been demonstrated that tFNAs are multifunctional DNA materials and can be used for a variety of applications, including biotherapeutics, drug delivery, bioimaging, and multiplexed sensing [67, 68]. In particular, tFNAs have found extensive use in the wound healing field [69, 70], as shown in Fig. 2. The main mechanisms and applications of tFNAs in wound healing are listed in Table 2.

In this section, the main applications of tFNAs in wound healing are described, including (a) as a delivery carrier; (b) regulation of cell proliferation and migration; and (c) pro-angiogenesis.

**Table 2:** Classification of tFNAs in wound healing and their mechanism

Types	Mechanism	Signaling pathway	Therapeutic agents		Results	Role	Ref
Tetrahedral DNA nanostructures	Promote cell proliferation	Wnt/β-catenin		None	CDKL1 $\uparrow$; β-catenin, Lef 1 ↓	Wound healing	[71]
Tetrahedral DNA nanostructures	Promote cell proliferation	Akt		None	VEGF, bFGF $\uparrow$ TNF-α, IL-1β $\downarrow$	Wounds scarless healing	[28]
Tetrahedral DNA nanostructures	Promote blood coagulation	Akt/Nrf2/HO-1		None	VEGF-A, NO $\uparrow$ TNF-α, IL -6 $\downarrow$	Diabetic wound healing	[72]
Tetrahedral DNA nanostructures	Inhibit apoptosis	PI3K-AKT-mTOR		None	CD31, VEGF $\uparrow$ Bcl-2, Bax Caspase-3 $\downarrow$	Wound healing	[73]
p@tFNAs	Antibacterial activity	RAS-ERK		Healing peptide	VEGFA, TGF-β, FGF2 $\uparrow$	As a carrier	[74]
Tetrahedral DNA nanostructures	Suppression of ROS production	Smad2/Smad3		None	Collagen I, fibronectin, α-SMA $\downarrow$	Fibrotic diseases therapy	[75]
tFNAs-Antibiotic Compound	Antibacterial activity	-		Ampicillin, erythromycin	IL-10 $\uparrow$ TNF-α, IL -6 $\downarrow$	As a drug carrier	[60]
Tetrahedral DNA nanostructures/wogonin complexes	Anti-inflammation	RhoA/ROCK		Wogonin	AGC, collagen II $\uparrow$ MMP, TNF-α $\uparrow$	Tissue regeneration	[76]

### As a delivery carrier

Bacterial infection presents a formidable obstacle in the clinical management of open wounds, attributable to the diverse range of pathogens involved [77, 78]. Despite the availability of broad-spectrum chemical antibiotics, challenges persist regarding their biosafety, accuracy, and efficacy. Moreover, the emergence of antibiotic-resistant bacteria has compounded the complexity of treating infected wounds [79, 80]. Consequently, the development of efficient delivery systems in wound healing assumes paramount importance in effectively addressing this issue [81–83]. In nanobiomedicine, drug delivery is a critical step. Many current drug carriers have some inherent limitations. For instance, hydrogels are a popular drug carrier, but some have poor biodegradability and may remain in the body for a long time, causing chronic inflammatory reactions or other adverse reactions [84–88]. Another type of nanomaterial, metal-organic framework materials, has been widely used as a drug delivery vehicle, but they are unstable in aqueous solutions, and their toxicity is not yet clear [89]. Compared to these drug carriers, tFNAs are composed of nucleic acids and are thus inherently biocompatible and biodegradable; their 3D geometry, surface chemistry, and dynamic functions are all programmable, making them unique as carriers in the treatment of wound healing [90, 91].

As highly loaded scaffolds that can regulate or maintain antimicrobial drug delivery, the use of DNA nanostructures as delivery vehicles for antimicrobial drugs has attracted interest [92–95]. Comparing DNA nanomaterials to more conventional drug delivery methods, such as organic nanomaterials or inorganic nanoparticles, DNA nanomaterials have demonstrated the capacity to precisely control the location and release of drug loading [96]. Due to their customizable functionality and biocompatibility, tFNAs have a wide range of applications in numerous fields [70]. In addition, tFNAs are a much smaller structure that can produce a similar result compared to the intricate DNA origami structure used as the carrier [44]. When tFNAs are encapsulated in tetrahedral nanostructures, they should have three properties to function in drug transport: (i) biological stability of DNA tetrahedra under physiological or pathological conditions to protect cargo; (ii) ease of cell membrane permeability to transport cargo; and (iii) structural integrity duration and cellular distribution for cargo release [97, 98].

**Fig. 3:**
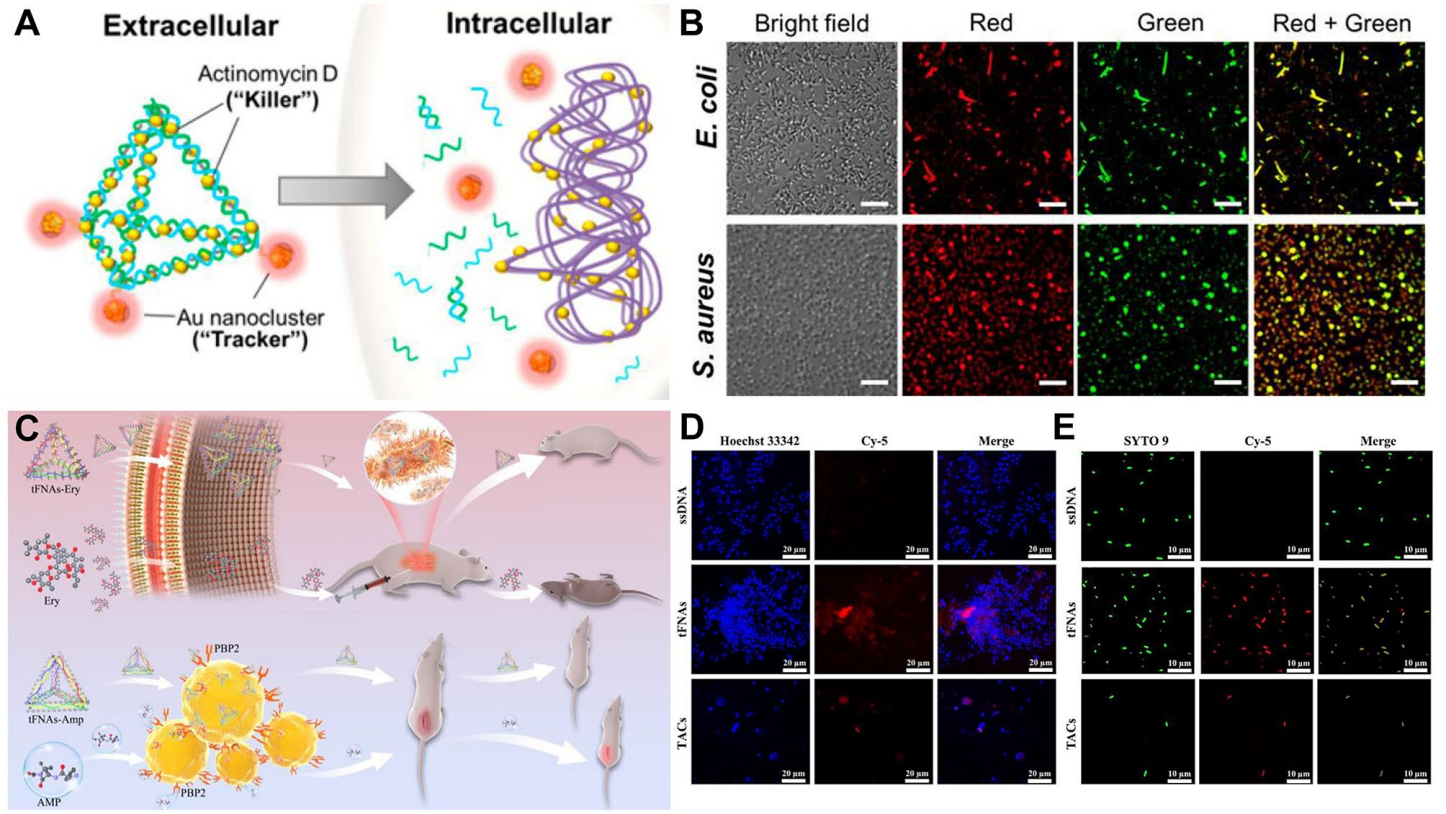
tFNAs as a delivery carrier with antibacterial activity. (**A**) Schematic representation of nanotheranostic DPAu/AMD synthesis. DP: DNA nanopyramid; AMD: Actinomycin D. Reprinted with permission from Ref. [59]. (**B**) DPAu/AMD-treated *E. coli* and *S. aureus* cells were stained with live/dead cell stain to validate the killing effect. *E. coli*: *Escherichia coli; S. aureus: Staphylococcus aureus.* Reprinted with permission from Ref. [59]. (**C**) tFNAs-Antibiotic Compound (TAC) improved the survival rate of severely infected mice and promoted the healing of local infections by the excellent delivery capability of tFNAs. tFNAs: tetrahedral framework nucleic acids. Reprinted with permission from Ref. [60]. (**D**) Confocal laser scanning microscopy images of bacterial uptake of tFNAs and TACs in MRSA at 90 min. Reprinted with permission from Ref. [60]. (**E**) Confocal laser scanning microscopy images of bacterial uptake of tFNAs and TACs in *E. coli* at 90 min. ssDNA: single-stranded DNA. Reprinted with permission from Ref. [60]

Recent studies have demonstrated that inorganic-based nanomaterials can cause toxicity, malfunction, and abnormalities in the mammalian cell system [99]. As a result, it is important to search for substitute materials that can complete the original task of delivering antimicrobial agents without harming the patient’s healthy cells. In their study, Setyawati et al. [59] delivered actinomycin D with tFNAs and integrated both antimicrobial and imaging capabilities into a single DNA-based entity called DPAu/AMD (Fig. 3A). To identify and evaluate the effectiveness of this nanotheranostic DNA structure, they further coupled red-emitting gold nanoclusters (Au NCs) on the apexes. It has been demonstrated that AMD binds to the dsDNA template and prevents DNA-dependent RNA synthesis, thereby disrupting the RNA elongation process [100]. DPAu/AMD was readily absorbed by *E. coli* and *S. aureus* and caused their death. The tFNAs were able to release the drug that would kill the bacterium by preventing RNA production once it had entered the cell and been broken down by DNase (Fig. 3B).

Tetrahedral nanostructures were shown to effectively penetrate cells due to their sharp corners [101], and by functionalizing tFNAs with ligands or medicines, tailored drug delivery was made possible [102, 103]. Researchers [60] have used two mouse models of systemic peritonitis and local wound infections to investigate the in vivo antimicrobial efficacy of a tFNA-antibiotic compound (TAC) (Fig. 3C). Due to increased bacterial antibiotic absorption and improved drug transport across cell membranes, TAC demonstrated a significant growth inhibitory effect on methicillin-resistant MRSA and *E. coli* in vitro. They conducted animal experiments using intraperitoneal and subcutaneous injections. According to the findings, TAC demonstrated good biosafety, increased the survival rate of mice that were severely infected, and aided in the healing of local infections (Fig. 3D-E).

**Fig. 4:**
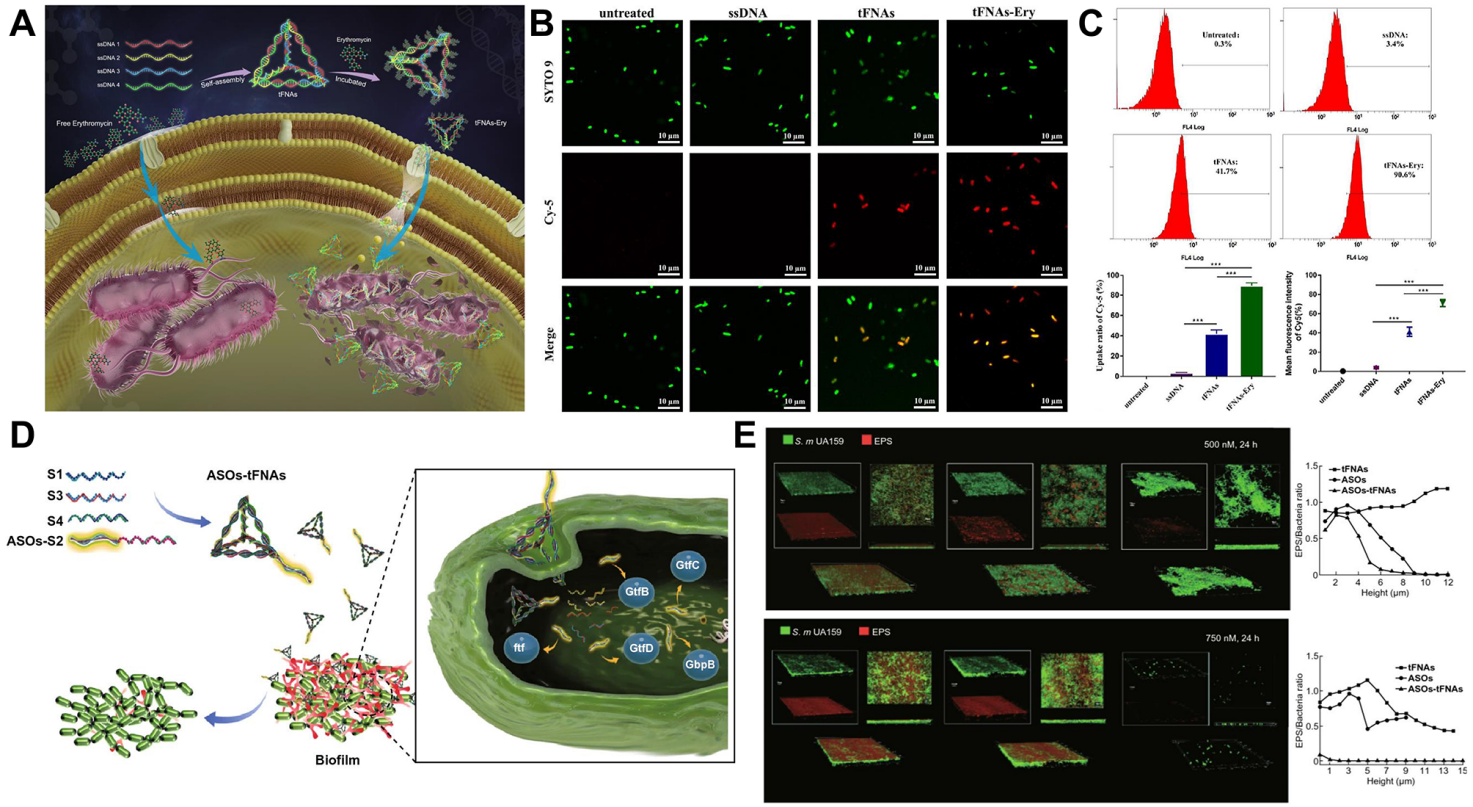
tFNAs as a delivery carrier with antibacterial activity. (**A**) tFNAs increase the erythromycin efficiency by delivering it more inside the cells. Ery: erythromycin. Reprinted with permission from Ref. [106]. (**B**) Confocal laser scanning microscopy images of bacterial uptake of tFNAs and tFNAs-Ery in *E. coli* at 90 min. Reprinted with permission from Ref. [106]. (**C**) Flow cytometry analysis of the uptake rates of *E. coli* incubated with ssDNA, tFNAs and tFNAs-Ery. Reprinted with permission from Ref. [106]. (**D**) Schematic representation of how tFNAs deliver ASOs to inhibit the formation of bacterial biofilms by targeting genes related to EPS synthesis. ASOs: antisense oligonucleotides. Reprinted with permission from Ref. [107]. (**E**) Dual-label imaging and three-dimensional visualization of EPS (red) and bacteria (green) in *S. mutans* biofilms after treatment with ASOs-tFNA at 500 nM and 750 nM. Reprinted with permission from Ref. [107]

Drug-resistant strains have developed quickly in recent decades as a result of improper medication usage [104]. However, few studies have used DNA nanomaterials as carriers of antibiotics to treat bacterial resistance to a certain degree. To reduce antibiotic efflux from antibiotic-insensitive bacteria, there may be a way to improve the delivery of antibiotics by endocytosis using tFNAs [105]. Sun et al. [106] investigated the delivery of erythromycin to *E. coli* (tFNAs-Ery) (Fig. 4A). Bacteria absorbed more erythromycin because of the tFNA carrier, which also helped the membrane stay stable. Additionally, it improved the passage of drugs across the membrane, increasing bacterial cell wall permeability and reducing drug resistance. tFNA-Ery allowed more erythromycin to effectively penetrate the cell, making it more effective than erythromycin alone due to the high transmembrane penetration capability of tFNAs. According to these findings, tFNAs help to increase the concentration of erythromycin in target cells (Fig. 4B-C).

Many chronic infections are caused by biofilm formation, which is a significant serious health problem. Since conventional therapies such as mechanical debridement, antibiotics, or biofilm destroyers are impotent against the biofilm’s capacity for self-protection and potent toxicity, the only workable solution is to stop biofilm formation in its earliest stages [108, 109]. Zhang et al. [107] developed a self-assembly framework nucleic acid delivery system that can deliver antisense oligonucleotides for targeting particular genes into bacterial cells (Fig. 4D). When delivered into the bacterial cells, the production of extracellular polysaccharide (EPS) and the biofilm thickness were both significantly decreased. Based on the results obtained, a concentration of 750 nM ASO-tFNA simultaneously inhibited the expression of gtfBCD, gbpB, and ftf and resulted in significant inhibition of EPS synthesis, thus reducing biofilm formation and virulence in the initial phase. A new nucleic acid-based nanomaterial with multitarget inhibition has a great deal of potential for treating persistent infections caused by biofilms (Fig. 4E).

It has been demonstrated that particular antisense peptide nucleic acids (asPNAs) target particular genes and repress transcription. Without a valid vector, however, few asPNA molecules penetrate bacterial cells [110]. Zhang and his coworkers [111] developed an efficient tFNA system for delivering asPNAs (P-TDNS). Although asPNA had some distinctive advantages, because it was not charged, it had difficulty penetrating bacterial cell walls. By efficiently delivering asPNA to drug-resistant bacterial cells and dramatically lowering the expression of the target gene, the engineered tFNA delivery system was able to successfully suppress cell growth (ftsZ). P-TDNA can pass through MRSA cell walls. Additionally, they had the ability to transport antisense antibiotics to specific genes, which efficiently suppressed their expression and, in a concentration-dependent way, hindered the proliferation of MRSA cells. The findings imply that tFNAs are a potential asPNA drug delivery system and an excellent method for delivering drugs to animal cells.

Cell-penetrating peptides have some significant problems, including inherent toxicity to both bacterial and mammalian cells. Moreover, it is currently costly and time-consuming to synthesize peptide-conjugated antisense oligonucleotides on a large scale. In the presence of cefotaxime, Readman et al. [61] developed and evaluated a DNA tetrahedral nanoparticle vector with a tailored anti-*bla*_CTX−M−group 1_ antisense peptide nucleic acid (PNA4) to break through the bacterial cell wall. Previous research on this anti-*bla*_CTX−M−group 1_ PNA demonstrated that it can partially restore cefotaxime sensitivity in strains with decreased resistance phenotypes and translationally limit the development of β-lactamase CTX-M-group 1 infield and clinical *E. coli* isolates [112]. PNA4 was added to the DNA tetrahedron vector structure, which resulted in a dose-dependent CTX-enhancing effect. When the DNA tetrahedron vector and PNA4 were present, the MIC (relative to CTX) of an *E. coli* field isolate containing the BlaCTX-M-3 plasmid dropped from 35 to 16 mg/L, indicating that a DNA tetrahedron was able to enter through the bacterial cell wall and deliver its payload, which was able to specifically attach to its target and suppress CTX-M protein production.

### Regulation of cell proliferation and migration

Cell proliferation and migration are necessary biological behaviors [113]. The inflammatory phase, the tissue formation phase, and the tissue reorganization and remodeling phase are the three main stages of the complicated processes involved in the healing of cutaneous wounds [114] and may involve a variety of cell types, including keratinocytes and fibroblasts. Collagen remodeling and new tissue growth are part of the tissue reorganization and remodeling phase, which results in the creation of a scar [115]. The ability of injured tissues and organs to perform normally might be impacted by poor posttraumatic wound healing.

**Fig. 5:**
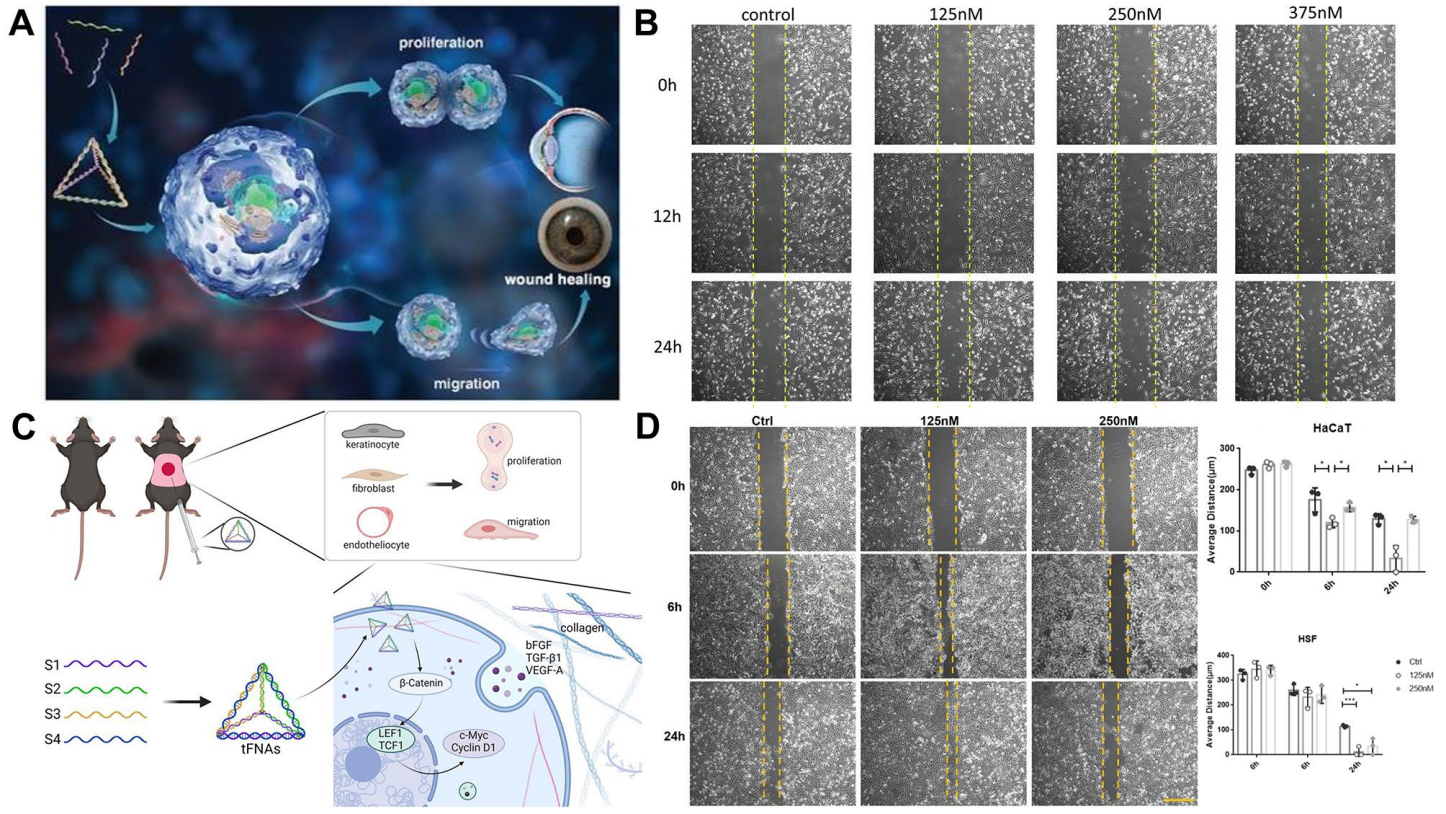
tFNAs promote regulation of cell proliferation and migration. (**A**) tFNAs promote corneal epithelial wound healing. Reprinted with permission from Ref. [25]. (**B**) An in vitro scratch wound healing assay detected the effect of tFNAs at different concentrations on the migration of human corneal epithelial cells (HCECs). Reprinted with permission from Ref. [25]. (**C**) Schematic diagram of the regulatory effects of tFNAs on TGF-β1-induced fibrogenesis via interaction with Smad2/Smad3 signals. Reprinted with permission from Ref. [28]. (**D**) Images of scratch tests on HaCaT cells treated with different concentrations of tFNAs at 0 h, 6 h, and 24 h. Reprinted with permission from Ref. [28]. (**E**) Statistical analysis of scratch tests on HaCaT cells and HSF cells. Reprinted with permission from Ref. [28]

The wound healing process for corneal damage is intricate and involves cell migration, cell death, and cell proliferation [116]. Once tFNAs are carried into cells, several biological processes can be impacted, including osteogenic differentiation, regeneration of nerve tissue, and anti-inflammatory and antioxidant activities [117, 118]. Moreover, tFNAs are simple to synthesize into eye drops. Taking corneal wound healing as an example, Liu et al. [25] explored the effect of tFNAs on wound healing and investigated the impact of tFNAs on HCEC migration and proliferation after corneal alkali burns. Interestingly, the experimental group was treated with tFNA eye drops prepared with normal saline solution (250 × 10^− 9^ M) eye drops. The findings demonstrated that tFNAs might quicken the migration and proliferation of HCECs in vitro. The biological function change in HCECs caused by tFNAs may be related to the increase in p38 and ERK1/2 phosphorylation levels (Fig. 5A). tFNAs can increase the transparency of the cornea following corneal burns and speed up the healing rate of the corneal wound. The wound healing effects of tFNAs on the corneal epithelium can be explained by their anti-inflammatory, antioxidant, and regenerative potential (Fig. 5B).

Regular ssDNAs are difficult to integrate into cells, while tFNAs can be abundantly taken up by caveolin-mediated endocytosis without the need for further delivery aids [119–121]. Earlier research has shown that tFNAs can stimulate cell migration and proliferation while also reducing inflammatory responses [122]. In this study, Zhu et al. [28] examined the effects of tFNAs on keratinocytes (HaCaT cell line) and fibroblasts (HSF cell line) in vitro and in vivo. After subcutaneous injection of 100 µL of 125 nM tFNAs, the results showed that in vitro, by activating the AKT signaling pathway, tFNAs can increase the migration and proliferation of HaCaT and HSF cells, as well as the secretion of growth factors by HSF cells, and suppress inflammatory responses in HaCaT cells. In addition, tFNAs had the power to prompt healing of the skin wound and decrease the formation of scars in vivo. These results suggest that tFNAs have great potential for clinical applications to lessen scarring and hasten the healing of wounds.

Chronic wounds do not heal as a result of decreased growth factor production, impaired angiogenesis, decreased cell proliferation and migration, ongoing inflammatory response, and abnormal collagen formation [123]. tFNAs may have an impact on many signaling pathways, including the Nrf2 and Wnt pathways, to encourage angiogenesis, hasten wound healing, and enhance healing quality [124, 125]. Wang et al. [27] investigated the healing mechanisms and therapeutic effects of tFNAs in diabetic wounds (Fig. 5C). The study showed that treatment with tFNAs under high glucose conditions increased cell migration and proliferation. In vitro, tFNAs also modulated the expression of growth factors. After subcutaneous injection of tFNAs, the in vivo test showed that tFNAs promoted collagen, capillary, and epidermal regeneration in addition to accelerating the healing of diabetic wounds. The Wnt pathway was also activated, and growth factor secretion was improved by tFNAs in diabetic wounds (Fig. 5D-E).

**Fig. 6:**
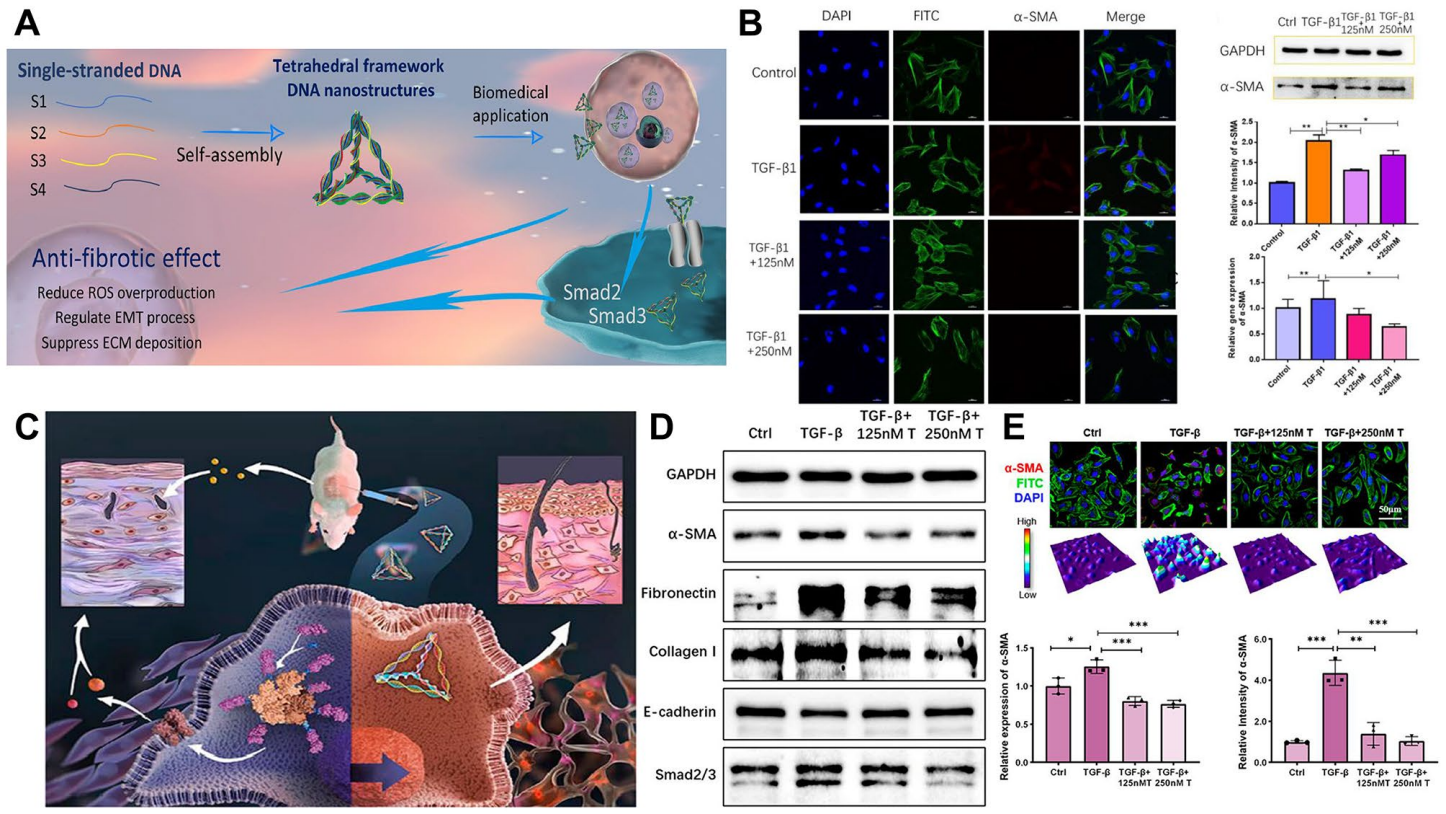
tFNAs promote regulation of cell proliferation and migration. (**A**) Schematic diagram of the regulatory effects of tFNAs on TGF-β1-induced fibrogenesis via interaction with Smad2/Smad3 signals. ROS: reactive oxygen species; EMT: epithelial-mesenchymal transition; ECM: extracellular matrix. Reprinted with permission from Ref. [75]. (**B**) TFNAs reduce TGF-β1-induced α-SMA expression in RLE-6TN cells. TGF-β1: Transforming growth factor beta 1. Reprinted with permission from Ref. [75]. (**C**) tFNAs reduce skin fibrosis by inhibiting the pyroptosis pathway. Reprinted with permission from Ref. [128]. (**D**) Western blotting (WB) analysis of the α-SMA, fibronectin, collagen I, E-cadherin, and Smad2/3 expression levels. Reprinted with permission from Ref. [128]. (**E**) Immunofluorescence (IF) micrographs of TGF-β- and tFNA-treated HaCaT cell 3D reconstruction diagrams and statistical analyses of IF and WB results for protein expression. Reprinted with permission from Ref. [128]

Under pathological circumstances, wound healing processes can become dysregulated and chronic, which can result in fibrosis [126]. Inflammation has been proposed to precede the development of fibrosis, and mounting research has revealed that several inflammatory mediators play a role in the fibrogenesis process [127]. tFNAs can regulate fibrotic processes and have a beneficial impact on the therapeutic management of fibrotic disease. Epithelial-mesenchymal transition (EMT), excessive extracellular matrix deposition, fibroblast proliferation, and transformation are regarded as the main pathogenic alterations that might cause fibrotic illnesses [127]. Zhang et al. [75] examined the antifibrotic effects of tFNAs on alveolar epithelial cells in lung tissue. With the stimulation of TGF-1, a cellular fibrosis model was created, and its underlying molecular mechanisms as well as the creation of ROS, ECM, and EMT with fibrogenesis were studied. ECM deposition and EMT are both important pathological mechanisms responsible for fibrosis development, as demonstrated in Fig. 6A, and it is crucial to inhibit the EMT process to treat fibrotic disorders. ROS overproduction and the TGF-1-induced EMT process were inhibited by tFNAs. Collagen I and fibronectin are two key molecules that influence the development of fibrosis, and tFNA therapy downregulates their expression. In addition, the WB results also demonstrated that tFNAs inhibit the expression of α-SMA. According to these findings, tFNAs may represent a new DNA nanomaterial that has the potential to control the EMT process (Fig. 6B).

When the degree or duration of skin injury surpasses the capacity of tissue repair, fibrosis predominates the healing process. The need for medications that effectively suppress skin fibrosis and lessen immunogenicity, inflammation, apoptosis, and pyroptosis is highlighted by the need that current approaches for treating skin fibrosis are inadequate and have adverse effects. Recently, Jiang et al. [128] examined the potential of tFNAs for the in vivo and in vitro treatment of skin fibrosis (Fig. 6C). According to the in vitro findings, the expression of proteins involved in the pyroptosis pathway was suppressed by tFNA therapy. tFNA therapy decreased the levels of inflammatory proteins and cytokines while inhibiting EMT, ECM deposition, and the Smad2/3 pathways. The skin thickness of mice was decreased by 33% after subcutaneous treatment with tFNAs along with relative decreases in fibrosis levels. It also ensured that the skin’s architectural integrity was maintained and that it was resistant to adverse external stimuli. The study found that tFNAs have the potential to cure disorders linked to pyroptosis and have a synergistic function in fibrosis therapy (Fig. 6D-E).

### Angiogenesis

Blood vessels are distributed throughout the body, carrying nutrition and oxygen and eliminating CO_2_ and waste to satisfy all kinds of physical needs. Early vascularization is therefore a defining characteristic of tissue repair, including bone regeneration and skin wound healing [129, 130]. Some wounds have a propensity to heal slowly, leading to the development of chronic wounds. Numerous factors can influence the chronicity of wounds, including circulatory disorders, diabetes mellitus, compromised immune function, and local pressure. These factors create a challenging environment for the wound, impeding the formation of new tissue and blood vessels and consequently causing delays in the healing process [131–133]. One of the main issues with wound healing is angiogenesis, which is a crucial mechanism by which cells at the wound site obtain oxygen and nutrients [134]. According to previous studies, tFNAs may impact a variety of biological behaviors in cells, including chondrocyte migration and proliferation, mesenchymal stem cell osteogenesis, and the antioxidant and anti-inflammatory capacities of macrophages [135]. Additionally, tFNAs encourage the release of vascular endothelial growth factor and basic fibroblast growth factor from the wound surface, which aids in wound healing.

**Fig. 7:**
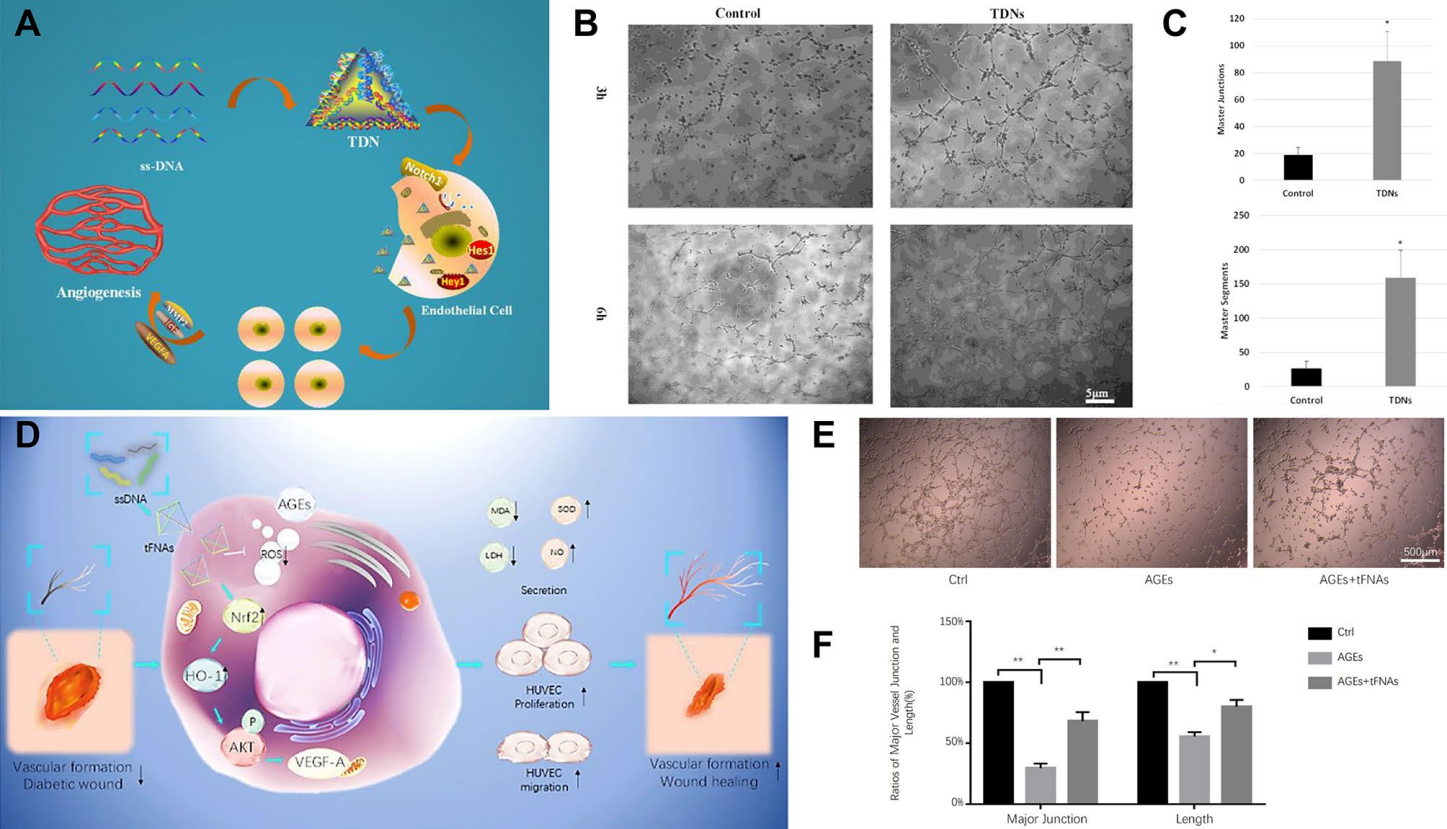
Angiogenesis-promoting effects of tFNAs. (**A**) tFNAs promote endothelial cell proliferation, migration, and angiogenesis via the Notch signaling pathway. TDN: tetrahedral DNA nanostructures. Reprinted with permission from Ref. [136]. (**B**) TDNs promoted tube formation of endothelial cells (ECs). Reprinted with permission from Ref. [136]. (**C**) Measurement of master junctions and master segments; Reprinted with permission from Ref. [136]. (**D**) Schematic diagram of the antioxidative and angiogenesis-promoting effects of tFNAs in diabetic wound healing. Reprinted with permission from Ref. [72]. (**E**) Tube formation assay. AGEs: advanced glycation end products. Reprinted with permission from Ref. [72]. (**F**) Semiquantification analysis of the major junctions and lengths of formed vessels. Reprinted with permission from Ref. [72]

For instance, Zhao et al. [136] showed that tFNAs may penetrate endothelial cells (ECs) and stimulate EC migration, tube formation, proliferation, and expression of angiogenic growth factors by triggering the Notch signaling pathway (Fig. 7A). Angiogenesis is a complex process in which endothelial cell-stimulated factors activate, proliferate, migrate, and reconstruct new blood vessels in vivo [137]. Consequently, EC migration and proliferation are crucial for angiogenesis. The RTCA test indicated that tFNAs could promote EC proliferation after 12 h of cultivation. With the increase in the concentration of tFNAs, the proliferation promotion effect was enhanced, and the results indicated that at 250 nmol L^− 1^, tFNAs could enter ECs and stimulate EC migration, proliferation, tube formation, and angiogenesis. The wound healing test indicated that tFNAs could significantly promote EC migration after 24 h. These findings suggest that tFNAs play an important role in angiogenesis promotion (Fig. 7B-C).

Neovascularization is essential for the healing of damaged tissue, as the formation of new blood vessels makes it easier to form granulation tissue and retain keratin-forming cells [138, 139]. A growing body of research suggests that tFNAs have advantageous impacts on the biological activities of cells, such as cell migration, proliferation, and anti-inflammatory and oxidant resistance qualities [140]. To determine whether tFNAs can encourage angiogenesis in diabetic wounds, Lin et al. [72] investigated the curative effect of tFNAs on diabetic mucosal wound healing and related angiogenesis in an in vivo diabetic animal model (Fig. 7D). A balance was achieved between oxidative and antioxidative factors in the tFNA regulation process. Anti-inflammatory and antioxidant indicators were increased. After cell ingestion, antioxidant and angiogenesis-promoting actions may be observed. After subcutaneous treatment with tFNAs, diabetic rats treated with tFNAs showed better healing ability of the ulceration. tFNAs were shown to accelerate epithelialization, vascularization, collagen deposition, and alignment to facilitate diabetic wound healing. According to the study, in the healing of diabetes, tFNAs can reduce inflammation, prevent oxidative damage, and promote angiogenesis (Fig. 7E-F).

**Fig. 8:**
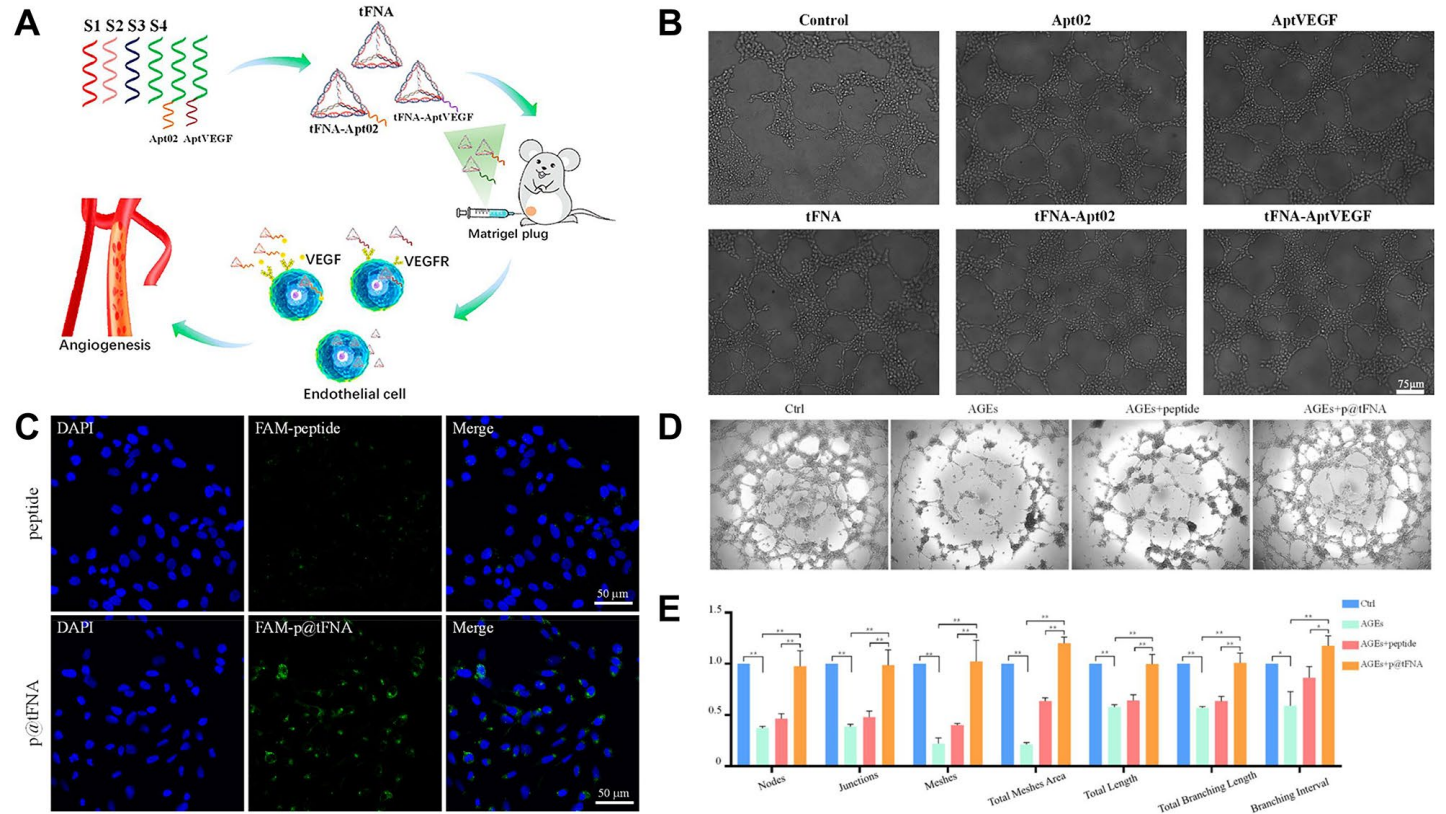
Angiogenesis-promoting effects of tFNAs. (**A**) Angiogenic aptamer-modified tFNAs promote angiogenesis in vitro and in vivo. Reprinted with permission from Ref. [124]. (**B**) Tube-formation assay for evaluating vascularization after treatment with nanoparticles. VEGF: Vascular endothelial growth factor. Reprinted with permission from Ref. [124]. (**C**) Cellular uptake of FAM-labeled peptides and FAM-labeled p@tFNA. Reprinted with permission from Ref. [74]. (**D**) Tube formation assay. p@tFNAs: tetrahedral framework nucleic acid (tFNA)-based peptide. AGEs: advanced glycation end products. Reprinted with permission from Ref. [74]. (**E**) Analysis of tube nodes, junctions, meshes, total meshes area, branching lengths and branching intervals of formed vessels. p@tFNAs: tetrahedral framework nucleic acid (tFNA)-based peptide; AGEs: advanced glycation end products. Reprinted with permission from Ref. [74]

Necrosis or the absence of new tissue is caused by a lack of new blood vessels in the material, ischemia in the center, and a discontinuous supply of nutrients and oxygen [141]. As a result, Zhao et al. [124] created aptamer-tFNA, tFNA-Apt02, and tFNA-AptVEGF by combining tetrahedral framework acid with two different angiogenic DNA aptamers. Both in vitro and in vivo studies were conducted to determine their impacts on angiogenesis (Fig. 8A). The tFNAs showed a higher capacity to stimulate angiogenesis when they were transformed with the aptamers Apt02 and AptVEGF. They encouraged the growth and migration of human umbilical vein endothelial cells (HUVECs), as well as the development of tubes, spheroid sprouts, and microvessels. It appears that aptamer-tFNA interactions are not only detrimental to their individual capabilities but also exhibit greater angiogenesis potential when combined (Fig. 8B).

Insufficient local angiogenesis can exacerbate the inflammatory response and lead to cell dysfunction, which in turn results in severe environmental conditions [142, 143]. Lipids, peptides, and proteins that stick to tFNAs shield them from their complicated environment and preserve their antioxidant capacity. Lin et al. [74] created p@tFNA, which is a tetrahedral framework nucleic acid (tFNA)-based peptide delivery system, to address the problems with therapeutic peptide stability and intracellular administration in diabetic wound healing. While tFNAs served as a frame and hollow interior space for the electrostatic adsorption of healing peptides, peptides were attached outside of tFNAs for wrapping. Peptides were more easily absorbed and remained stable in the tFNA frame, while the peptide shielded the tFNAs from DNase. Through ERK1/2 phosphorylation, p@tFNA assisted in overcoming the angiogenesis barriers caused by AGEs. After subcutaneous treatment with tFNAs, the antioxidation activity exhibited by p@tFNA also paved the way for diabetic wound healing. As a result, both in vitro and in vivo, p@tFNA can enhance angiogenesis and the healing of diabetic wounds. p@tFNA promoted the formation of blood vessels, collagen deposition, and epidermal covering, thus laying the foundation for improved regenerative effects in skin defects (Fig. 8C-E).

## Biocompatibility and biosafety

To strengthen the further antibacterial applications of tFNAs, it is necessary to evaluate their biosafety. The programmability, biocompatibility, and biostability of tFNAs have led to their widespread usage in biomedicine. (i) Programmability: As previously mentioned, DNA nanostructures range in size from a few nanometers to micrometers and have diverse shapes, giving tFNAs variable cellular absorption pathways and targeting and traffic differently in animal models [144, 145]. (ii) Biosafety: The results of the available study indicate that tFNAs demonstrate good biosafety. The remainder can be broken down in the liver or circulation without posing a long-term safety risk when using tFNAs that are rationally designed to quickly reach the target location and demonstrate their efficacy [146]. Because of the great biocompatibility and intrinsic low toxicity of tFNAs from ssDNA and dsDNA, they can be used in vivo, including in cells, tissues, and organisms [147]. (iii) Biostability: Densely packed tFNAs typically demonstrate superior resistance to nuclease degradation since they have much less exposed free ssDNA [148]. Long-term drug release, targeted drug administration, and other applications are made possible by increased stability in the physiological environment and an extended retention period in animal models [149].

## Summary and outlook

In recent years, the new field of tFNAs for wound healing applications has shown versatile potential and attracted increasing interest. In this review, we discuss the current use of tFNAs in wound healing. First, we summarize the advantages of tFNAs in promoting wound healing, including simple synthesis and high stability, which provide a foundation for the subsequent construction of drug-loaded imaging and targeted therapy. Then, we introduce the mechanism and application of tFNAs in wound healing. At the same time, tFNAs, combined with other materials such as antibacterial agents and antisense peptide nucleic acids, have superior antibacterial properties and target specific genes, thereby achieving efficient antibacterial activity. The incorporation of small-molecule drugs and bioactive factors into tFNAs has facilitated the inhibition of inflammation, decreased infection, accelerated wound closure, and reduced scar formation.

Hence, tFNAs in wound healing applications hold promising prospects. First, in facilitating wound regeneration, tFNAs can target specific genes or signaling pathways to enhance key biological processes involved in wound healing, such as cell proliferation, migration, and differentiation. This accelerates wound repair and promotes the formation of new tissue. Then, in anti-inflammatory and anti-infective potency, tFNAs can be utilized to suppress inflammatory responses and reduce the risk of infection. By regulating the expression of inflammation-related genes and intervening in the growth of pathogenic microorganisms, tFNAs have the potential to lower the likelihood of wound infections, improving wound cleanliness and health. Third, in the regulation of cell proliferation and migration, tFNAs furnish a three-dimensional architectural framework that facilitates the proliferation and motility of cells. Functioning as a dynamic scaffold, tFNAs orchestrate the orchestrated expansion of cells within injured tissue, stimulating neovascularization and tissue regeneration. By regulating collagen synthesis, cell migration, and extracellular matrix remodeling, tFNAs hold promise in improving wound healing outcomes and reducing the risk of scar formation. Fourth, in fostering angiogenesis, tFNAs can stimulate endothelial cell proliferation and the expression of angiogenesis-related factors, facilitating the formation of new blood vessels. This enhances oxygen and nutrient supply to the wound, boosting blood flow and expediting the wound healing process. Last but not least, in personalized therapy, with their tunable and customizable nature, tFNAs can be tailored to meet the specific needs of individual wounds. By selecting appropriate functional nucleic acid sequences and administration methods, precise treatments can be achieved for different wound types and stages, maximizing efficacy and personalized therapeutic outcomes.

In conclusion, tFNAs exhibit vast application prospects in wound healing. Further research and development efforts will optimize tFNA design and administration strategies, driving their widespread clinical implementation and providing more effective and personalized solutions for wound management.

With growing interest in tFNAs as smart platforms for biological applications, tFNAs are regarded as superior agents for therapeutic wound healing. However, there are still several challenges to overcome before these framework materials can be used for clinical diagnosis and treatment. Therefore, this paper also provides a corresponding perspective based on the authors’ insights.

The improvement of the preparation and application of tFNAs in the future can be considered from the following aspects. (1) The safety of tFNAs cannot be ignored. Regarding biomedical applications, the materials chosen by researchers must be nontoxic and harmless, paying special attention to side effects, metabolism, and possible immune responses of the body. The therapeutic mechanism and toxicity of tFNAs in wound healing should be studied by large-scale clinical trials in the future. In particular, further experiments are needed to elucidate the interaction of tFNAs with wound cells, as well as the degradation, metabolism, and clearance pathways of tFNAs. Universal and complete screening and evaluation criteria to detect toxicity also need to be established. (2) The physicochemical properties of tFNAs should be studied more deeply, and their combination with other materials should be considered to obtain better biocompatibility and better wound healing effects while minimizing side effects on normal tissues. (3) The design of tFNAs can be enhanced in various fields, such as materials science, nanotechnology, pharmacy, and clinical medicine. Researchers need to expand the field of application and focus on solving practical problems. (4) Clinical translation has always been a challenge for nanomaterials. A method to industrialize the production of tFNAs while ensuring that the products are stable, safe, and controllable is a major problem that researchers need to overcome.

In our opinion, framework nanomaterials have great potential for use in wound healing and regeneration. Although great research progress has been made based on tFNAs, there is still much work to be done on tFNAs in skin wound healing applications compared to other fields. In conclusion, we hope that the current challenges will encourage and trigger more efforts in this emerging field of research. With an increasing number of explorations from all over the world, we believe that novel framework materials based on tFNAs will have bright prospects in the future.

## Data Availability

Not applicable.
